# Translating proteome and transcriptome dynamics of periodontal ligament stem cell-derived secretome/conditioned medium in an in vitro model of periodontitis

**DOI:** 10.1186/s12903-024-04167-z

**Published:** 2024-03-27

**Authors:** Han Na Suh, Ju Young Ji, Jung Sun Heo

**Affiliations:** 1 Korea Institute of Toxicology, 30 Baekhak1-Gil, Jeongeup, Jeollabuk-Do 56212 South Korea; 2 Department of Maxillofacial Biomedical Engineering, College of Dentistry, Kyung Hee University, 26 Kyunghee-Daero, Dongdaemun-Gu, Seoul, 02447 South Korea

**Keywords:** Periodontal ligament stem cells, Secretome/conditioned medium, Osteogenic differentiation, Proteomic and transcriptomic profiling

## Abstract

**Background:**

Periodontal ligament stem cells (PDLSCs) have been proposed as therapeutic candidates in periodontal diseases and periodontium defects. Paracrine factors of PDLSCs, namely, secretome, can contribute to tissue regeneration comparable to direct stem cell application. This study explored restoration effects of PDLSC-derived secretome/conditioned medium (PDLSC-CM) on PDLSCs themselves in an inflammatory microenvironment and identified its action mechanisms using proteomics and transcriptomic profiling.

**Methods:**

PDLSC-CM was prepared from cells under healthy culture conditions. Mass spectrometry and liquid chromatography–tandem mass spectrometry (LC–MS/MS) were then performed to analyze the PDLSC-CM proteome. Osteogenic differentiation of PDLSCs under inflammatory conditions or in the presence of PDLSC-CM was then characterized in assays of alkaline phosphatase activity, intracellular calcium levels, protein expression of osteogenic markers, and matrix mineralization. Furthermore, the transcriptomic profile was assessed to identify significantly enriched signaling pathways and associated molecular networks by RNA sequencing.

**Results:**

LC–MS/MS proteomics identified a total of 203 proteins and distinguished 187 significant protein changes in PDLSC-CM compared to control-CM. LPS-treated PDLSCs significantly attenuated osteogenic differentiation. When PDLSCs were treated with PDLSC-CM alone, their osteogenic activity was significantly upregulated compared to the control group. Moreover, the LPS-impaired osteogenesis of PDLSCs was reconstituted by PDLSC-CM treatment. RNA sequencing revealed 252, 1,326, and 776 differentially expressed genes in the control vs. LPS, control vs. PDLSC-CM, and LPS vs. LPS + PDLSC-CM groups, respectively.

**Conclusion:**

This study suggest that PDLSC-CM restores the osteogenic potential of PDLSCs in an inflammatory environment through secretory functions representing potential repair and regenerative mechanisms.

**Supplementary Information:**

The online version contains supplementary material available at 10.1186/s12903-024-04167-z.

## Introduction

Extensive research has suggested that stem cell therapy is a viable treatment for managing various medical disorders and rehabilitating damaged tissues. However, the direct administration of stem cells faces multiple clinical risks, such as immune rejection and tumor formation [1]. In particular, mesenchymal stem cells (MSCs) cultured in vitro can develop modifications in cellular adhesion molecules, eventually resulting in the loss of cell-homing capacity and inappropriate stem cell migration [2]. These issues have recently encouraged indirect approaches in stem cell therapy that can be suggested as substitutes for MSC engrafting. Previous studies have demonstrated that the therapeutic potential of stem cells is partially attributed to their secretion of paracrine factors, including growth factors, cytokines, and many other bioactive molecules (i.e., soluble proteins, nucleic acids, lipids, and extracellular vesicles) into their surrounding medium, which is then called conditioned medium (CM). These stem cell-secreted molecules, collectively known as the secretome, have been described to influence numerous cellular activities and to contribute to tissue repair and regeneration, corresponding to the therapeutic effects of stem cell transplantation [3–5].

Periodontal ligament stem cells (PDLSCs), a type of dental MSC, are among the most promising MSC populations for periodontal regeneration [6]. PDLSCs can also differentiate into osteogenic, chondrogenic, adipogenic, and neurogenic cell lineages, similar to nondental MSCs [7–9]. In addition to these PDLSC characteristics, their therapeutic and homeostatic capacity in periodontal tissues, including alveolar bone, cementum, periodontal ligament, and gingival tissue, is exhibited by their secreted paracrine molecules, i.e., the secretome [10]. PDLSC-derived secretome/CM has currently demonstrated therapeutic effects, such as periodontal regeneration, anti-inflammatory, chondrogenic, and osteogenic regulation in various in vivo and in vitro experimental models [6, 11–15]. However, to understand the therapeutic role of PDLSC-CM, the identifying the key factors therein and the molecular mechanisms by which such biomarker proteins implement therapeutic utility needs to be highlighted. Several studies with respect to therapeutic proteins in the PDLSC-CM have been searched through some of the current proteomic techniques with cytokine profiles, immunological assays, and western blotting toward a wide range of known proteins [6, 10, 16, 17]. As proteomic technology has advanced, shotgun proteomic approaches such as liquid chromatography-tandem mass spectrometry (LC–MS/MS) have been also employed to explore unknown and unique proteins in the secretome from other type of dental tissue-stem cells [18]. Thus, proteomics-based approaches with PDLSC-CM continue to predict the key therapeutical proteins, but a substantial understanding of the biological action and molecular mechanism of this secretome/CM under individual experimental or clinical condition is still required. Together with proteomics, transcriptomic profiling utilizing RNA sequencing technology has strengthened the therapeutic potential of stem cell-derived CM exhibiting the molecular dynamics with global gene expression and specific signaling pathways in various biological systems [19–21]. To develop novel approaches of PDLSC-CM treatment, employing multiple omics technologies may provide a better understanding of the functional components of PDLSC-CM.

This study investigated the restorative effects of PDLSC-CM on the osteogenic capacity of PDLSCs in an inflammatory environment. To certify this hypothesis, we employed (1) an LC–MS/MS proteomics to classify the protein components of PDLSC-CM and (2) an RNA sequencing transcriptomics to validate the molecular dynamics and the potential signaling pathways for protective and regenerative functions of PDLSC-CM. These findings provide a platform for developing PDLSC-CM strategy as a new therapeutic option to overcome periodontal degenerative diseases.

## Materials and methods

### Periodontal ligament stem cell culture

Human PDLSCs were purchased (CELPROGEN, CA, USA) and cultured in α-Minimum Essential Medium (α-MEM, Gibco-BRL, Gaithersburg, MD, USA) containing 10% fetal bovine serum (FBS, Gibco-BRL) as previously described [22]. PDLSCs at passages 4–6 were used for all experiments. To perform each experiment, cells were cultured in an osteogenic medium (α-MEM containing 5% FBS, 50 μg/ml ascorbic acid, 1 μM dexamethasone, and 3 mM β-glycerophosphate). For the experiments involving PDLSC-CM and LPS (from *Porphyromonas gingivalis*) treatment, cells were divided into four groups: osteogenic medium (Control), osteogenic medium + LPS (5 μg/ml) (LPS), PDLSC-CM, and LPS + PDLSC-CM. For the PDLSC-CM and LPS + PDLSC-CM groups, PDLSC-CM was supplemented with the osteoinductive factors mentioned above, corresponding to the composition of osteogenic medium. Culture media were changed every 2 days. All reagents and laboratory consumables were obtained from the Sigma Chemical Company (St. Louis, MO, USA) and SPL Lifescience (Pocheon, Korea), respectively.

### Preparation of conditioned medium

PDLSC-CM was prepared from cells grown under healthy culture conditions. PDLSCs were cultured in 100-mm cell culture dishes until they reached 70% confluency and were then washed with phosphate-buffered saline (PBS), after which they were cultured in serum-free α-MEM without FBS and antibiotics for 48 h. Culture supernatants were then collected and centrifuged at 3,000 rpm for 5 min to remove cell debris. The resulting medium was defined as PDLSC-CM for all experiments. In LC–MS/MS analysis, control medium (as Control) was obtained by collecting the culture medium (serum-free α-MEM) from culture dishes without cells after 48 h of incubation.

### Protein digestion

Prior to digestion, proteins were processed using filter-aided sample preparation (FASP) on a Microcon 30 K centrifugal filter device (Millipore, Billerica, MA, USA) and reduced with Tris(2-carboxyethyl)phosphine (TCEP) at 37℃ for 30 min [23]. Each sample was then alkylated with iodoacetic acid (IAA) at 25℃ for 1 h in the dark and washed with lysis buffer and 50 mM ammonium bicarbonate (ABC). The proteins were then digested with trypsin at 37℃ for 18 h. The digested peptides were then desalted using C18 spin columns (Harvard Apparatus, Holliston, MA, USA) and eluted with 80% acetonitrile in 0.1% formic acid.

### LC–MS/MS analysis

LC–MS/MS analysis was performed according to a previous report [23]. Briefly, the digested peptides were resuspended in 0.1% formic acid and analyzed using a Q-Exactive Orbitrap hybrid mass spectrometer (Thermo Fisher Scientific, Waltham, MA, USA) with an Ultimate 3000 system (Thermo Fisher Scientific, Waltham, MA, USA). We used a 2 cm × 75 μm ID trap column packed with 3 μm C18 resin and a 50 cm × 75 μm ID analytical column packed with 2 μm C18 resin to separate peptides according to their hydrophobicity. Data-dependent acquisition was performed, and the top 10 precursor peaks were selected for fragmentation. Ions were scanned in high resolution (70,000 in MS1 and 17,500 in MS2 at m/z 400), and the MS scan range was 400–2,000 m/z at both the MS1 and MS2 levels. Precursor ions were fragmented with 27% normalized collisional energy. Dynamic exclusion was adjusted to 30 s. Each MS/MS raw files were assessed using Proteome Discoverer™ software (ver. 2.5), and the Homo sapiens database was downloaded from Uniprot. The workflow of proteome data analysis included a peptide-spectrum match (PSM) validation step and SEQUEST HT as a database search algorithm. Cut-offs below a false discovery rate (FDR) of 1% were adopted, and sequences were filtered to peptides containing at least 6 residues. The relative amount of the proteins among samples was calculated by label-free quantitation.

### Alkaline phosphatase activity

To evaluate the osteogenic differentiation of PDLSCs, alkaline phosphatase (ALP) activity was evaluated as described in a previous study [22]. In brief, the total protein was extracted, and its concentration was determined. Then, 200 μl of p-nitrophenylphosphate (pNPP) was added to each sample and incubated for 30 min at 37 °C. The mixture was then stopped with the addition of 3 M NaOH, and the optical density was measured using a spectrophotometer at 405 nm. The ALP activity was calculated as mM/100 μg of protein.

### Intracellular calcium quantification assay

The intracellular calcium level was assessed according to our previous report [22]. Briefly, cells were cultured in every experimental condition for 7 days, and the intracellular calcium concentration was measured using a calcium assay kit (BioAssay Systems, Hayward, CA, USA) according to the manufacturer’s instructions. The absorbance was then evaluated at 612 nm. The calcium content was calculated as mg/100 mg of protein.

### Alizarin Red S staining

To evaluate calcium deposits in cell cultures, Alizarin Red S Staining was performed according to our previous study [22]. The cells were washed with PBS and fixed in 4% paraformaldehyde for 15 min after a 14-day incubation. The cells were then rinsed three times with PBS and stained with 2% Alizarin Red S solution (pH 4.2) for 5 min at room temperature. The stained cells were washed and visualized using a light microscope. The stained area was measured with Image J software (National Institutes of Health, Bethesda, MD, USA).

### RNA extraction and real-time reverse transcription polymerase chain reaction

Real-time reverse transcription polymerase chain reaction (RT-PCR) was performed after RNA extraction and cDNA synthesis to validate RNA sequencing results as previously described [22]. Real-time RT-PCR was performed according to the instructions provided with the QuantiTect SYBR Green PCR kit (Qiagen) with an iCycler iQ Multi-Color Real-Time Detection System (Bio-Rad). The thermal cycling conditions were 95 °C for 30 s, 95 °C for 5 s, 55 °C for 30 s, and 72 °C for 30 s for 30 cycles. The primers were as follows: 5′-CGCTTCGATGACTGGTACCT-3′ (sense) and 5′-TAGGCCTCCAAGGACTGGAA-3′ (antisense) for endoplasmic reticulum (ER) degradation-enhancing alpha mannosidase-like protein 2 (*EDEM2*); 5′-CAGCTCTTTCCTCCAACCCT-3′ (sense) and 5′-GAAATTCCCGGAGCTCCAGA-3′ (antisense) for C-X-C motif ligand 3 (*CXCL3*); 5′-CTGCACAGATGAGAGACAAATTCC-3′ (sense) and 5′-GAAGCTGCAAAGATCCCAATG-3′ (antisense) for interleukin 11 (*IL11*); 5′-CTGGGACAGCGCCACATTCGCCGGAGGCGG-3′ (sense) and 5′-TCCGCAGAAAGCAGCCATAGGGGGTAGGCT-3′ (antisense) for a disintegrin and metalloproteinase 15 (*ADAM15*); 5′-TGTTAAGGCCATAGCTGCGT-3′ (sense) and 5′-TCGCACAGACACCTGGAAAA-3′ (antisense) for mohawk homeobox (*MKX*); 5′-CGCCTCTTCTTATCAAGCTCGTG-3′ and (sense) and 5′-GAAGCTGTCGTAATTCTGCCAGG-3′ (antisense) for phosphoinositide-3-kinase regulatory subunit 1 (*PIK3R1*); and 5′-GCTCTCCAGAACATCATCC-3′ (sense) and 5′-TGCTTCACCACCTTCTTG-3′ (antisense) for glyceraldehyde-3-phosphate dehydrogenase (*GAPDH*).

### Western blot analysis

The extracted protein samples were separated by sodium dodecyl sulfate (SDS)-polyacrylamide gel electrophoresis (SDS-PAGE) and transferred electrically to polyvinylidene difluoride (PVDF) membranes. To identify the osteogenic differentiation of PDLSCs, osteocalcin (OCN), osterix (OSX), and Runt-related transcription factor 2 (RUNX2) protein levels were analyzed using primary and secondary antibodies (goat anti-rabbit immunoglobulin G (IgG) and goat anti-mouse IgG conjugated to horseradish peroxidase). The antibodies were obtained from Santa Cruz Biotechnology (Santa Cruz, CA, USA) and Cell Signaling Technology (MA, USA).

### Library preparation and sequencing

RNA sequencing was performed as described previously [24]. In brief, total RNA was extracted from PDLSCs cultured under different conditions, and the 500 ng of total RNA was prepared. The sequencing library was constructed using a QuantSeq 3′ mRNA-Seq Library Prep Kit (Lexogen, Inc., Austria) according to the manufacturer’s specifications. The library was amplified to add the complete adapter sequences required for cluster production. To sequence the libraries, they were mixed, denatured with NaOH to achieve single-stranded DNA, and then sequenced using an Illumina NextSeq 500 (Illumina, Inc., USA).

### Data analysis

The reads generated in the RNA-seq data were aligned to a Bowtie2 index in which the reference genome and gene model annotation files (obtained from the Gene Expression Omnibus [GEO] website) were used for annotation [25]. Differentially expressed genes (DEGs) were assessed based on counts from unique and multiple alignments using the coverage command in Bedtools [26]. The RC (read count) data were obtained from the quantile normalization using the Bioconductor software package EdgeR [27]. The analyses of gene classification, ontology, and Kyoto Encyclopedia of Genes and Genomes (KEGG) pathways were processed by DAVID (http://david.abcc.ncifcrf.gov/). Data extractions and image visualizations were accomplished using ExDEGA (Ebiogen Inc., Korea). The protein–protein interaction (PPI) networks were constructed using STRING v3.8.2. A PPI score of > 0.9 (*P* < 0.05) was considered significant. The PPI networks were imaged using Cytoscape software (http://www.cytoscape.org).

### Statistical analysis

The data are presented as means ± SD (n ≥ 3) using the Student’s t-test. One-way analysis of variance was applied for multiple comparisons (Duncan’s multiple range test). A *P*-value < 0.05 was considered statistically significant. The figures shown are representative of the data.

## Results

### Protein expression of PDLSC-secretome/conditioned medium

**Fig. 1:**
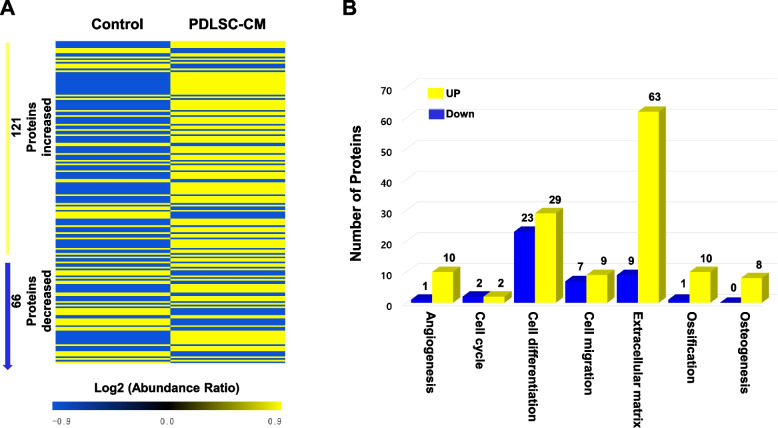
Proteomics analysis of proteins from PDLSC-CM. **A** Hierarchical clustering of significantly differentially expressed proteins. **B** Numbers of up- and downregulated proteins and principal components analysis in PDLSC-CM compared to those in control-CM (fold change ≥ 2 and *P*-value < 0.05). Yellow color represents upregulation; blue color represents downregulation

An LC–MS/MS proteomics approach was first performed to identify differentially abundant proteins in PDLSC-CM compared to control-CM (serum-free α-MEM). Among the 203 total proteins detected (Supplementary data 1), we distinguished 187 significant protein changes according to a fold change cut-off of ≥ 2 and a *P*-value < 0.05 (Supplementary data 2). Of these secreted proteins, 121 were present in increased levels in PDLSC-CM compared to control-CM, and 66 were present in decreased levels (Fig. 1A). We grouped these abundantly secreted proteins into broad categories based on various cellular activities, including angiogenesis (11 proteins), cell cycle (4 proteins), cell differentiation (52 proteins), cell migration (16 proteins), extracellular matrix (ECM) (72 proteins), ossification (11 proteins), and osteogenesis (8 proteins) (Fig. 1B). The top 10 PDLSC-secreted proteins were further classified and are listed in Table 1. Among these paracrine proteins, key components in the ECM including fibronectin and type I collagen were the abundantly secreted proteins from PDLSCs; it is known to participate in the bone formation and periodontal tissue regeneration [28–30]. In addition, keratin, one of major cytoskeleton related to stem cell proliferation and osteogenic differentiation of dental tissue-stem cells [31, 32], found at large number of peptides. Based on these proteomics profiling results, PDLSC-CM was determined to contain an extensive array of proteins that are potential candidates for regulating PDLSC activity and stimulating periodontal tissue regeneration.

**Table 1:** Top 10 abundantly secreted proteins in PDLSC-CM

Accession	Protein description	Peptides no	Abundance ratio	Coverage [%]
P02751-1	Isoform 1 of Fibronectin (FN1)	68	56.037	42
P02751	Fibronectin (FN1)	67	4.351	41
P02452	Collagen alpha-1 (COL1A1)	52	23.949	56
A0A087WTA8	Collagen alpha-2 (COL1A2)	41	26.429	46
P04264	Keratin, type II cytoskeletal 1 (KRT1)	34	0.033	46
A0A1B0GVI3	Keratin, type I cytoskeletal 10 (KRT10)	31	0.025	56
P35527	Keratin, type I cytoskeletal 9 (KRT9)	29	0.037	54
P07996	Thrombospondin-1 (THBS1)	29	40.681	29
P35908	Keratin, type II cytoskeletal 2 epidermal (KRT2)	27	0.014	47
P13647	Keratin, type II cytoskeletal 5 (KRT5)	25	0.015	38

### PDLSC-CM alleviates the inhibitory effect of LPS on the osteogenic differentiation of PDLSCs

**Fig. 2:**
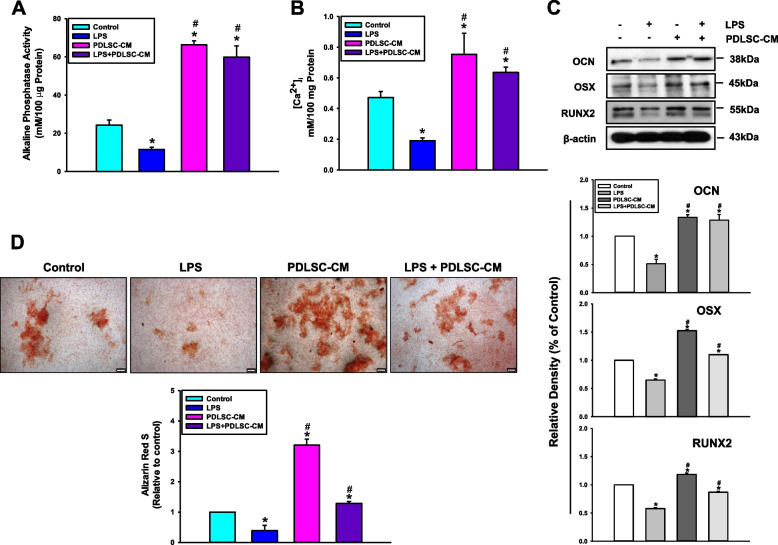
Effects of LPS and PDLSC-CM on the osteogenic differentiation of PDLSCs. Cells were cultured with LPS, PDLSC-CM, or LPS + PDLSC-CM for 7 or 14 days, and (**A**) ALP activity, **B** [Ca^2+^]_i_, and (**D**) Alizarin Red S staining (Scale bar, 200 μm) were assessed as described in the Materials and Methods. The panels (bars) denote a quantitative analysis of alizarin red S using image analysis software. The values are presented as the means ± SD (*n* = 5, *P < 0.05 *vs*. the control value; ^#^*P* < 0.05 *vs*. the LPS value at each time point). **C** The protein levels of OCN, OSX, and RUNX2 were analyzed by western blot after 7 days of osteogenic induction. The X-ray films of western blot were cropped, and full-length X-ray films are presented in Supplementary data 5. The panels (bars) represent the mean ± SD for each condition determined from densitometry relative to β-actin (*n* = 3, *P < 0.05 *vs*. the control value; ^#^*P* < 0.05 *vs*. the LPS value at each time point)

We next verified whether the secretome/PDLSC-CM can regulate the differentiation potential of PDLSCs in the context of an in vitro inflammatory microenvironment. Under LPS-induced inflammatory conditions, the osteogenic differentiation of PDLSCs was significantly inhibited, as shown by the ALP activity (Fig. 2A); furthermore, the intracellular calcium levels ([Ca^2+^]_i_) (Fig. 2B), the protein expression levels of osteogenic factors such as OCN, OSX, and RUNX2 (Fig. 2C), and extracellular calcium deposits (Fig. 2D) were downregulated in the LPS-treated group. However, when individual cell cultures were treated with PDLSC-CM, the osteogenic activity of the PDLSCs was markedly upregulated compared to the control group. Moreover, the application of PDLSC-CM relieved the decrease in the osteogenic differentiation of PDLSCs under the LPS-stimulated inflammatory conditions. These findings suggest that PDLSC-CM represents a functional formulation of the PDLSC secretome that can enhance the osteogenic differentiation potential of PDLSCs and could be considered a cell-free therapeutic resource for periodontal tissue repair and regeneration.

### DEGs and Gene Ontology (GO) enrichment analysis

**Fig. 3:**
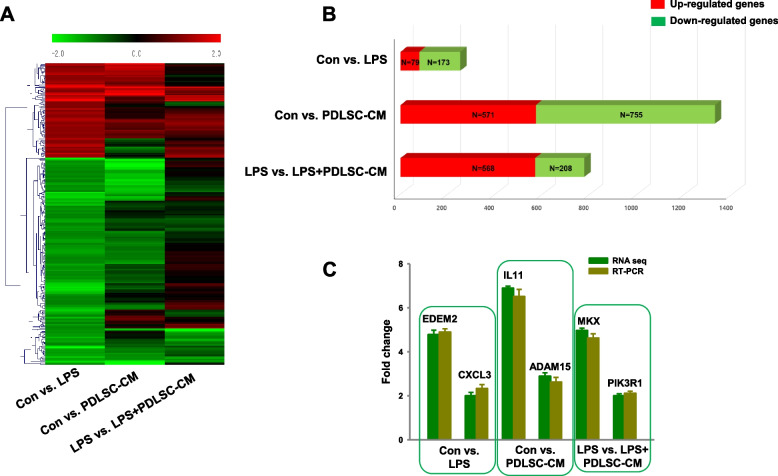
RNA sequencing analyses of PDLSCs cultured with LPS, PDLSC-CM, or LPS + PDLSC-CM. **A** Heatmap of significantly differentially expressed genes. **B** Numbers of up- and downregulated genes in cells. Red color represents upregulation; green color represents downregulation. **C** Real-time RT‑PCR validation of the expression levels of randomly selected DEGs

We then distinguished the DEGs in the control vs. LPS, control vs. PDLSC-CM, and LPS vs. LPS + PDLSC-CM groups by RNA sequencing analysis (with fold changes ≥ 2 and *P*-values < 0.05 representing significant differences), resulting in the identification of 252 (79 upregulated and 173 downregulated), 1,326 (571 upregulated and 755 downregulated), and 776 (568 upregulated and 208 downregulated) DEGs, respectively (Fig. 3A and B, Supplementary data 3). In addition, 290 of common genes (5 upregulated, 2 downregulated, and 283 contra-regulated) among the different conditions were identified (Supplementary data 4). The transcript profiling was then validated by real-time RT-PCR, which verified that the mRNA expression levels of EDEM2, CXCL3, IL11, ADAM15, MKX, and PIK3R1 were consistent with the RNA-seq data in the different comparisons (Fig. 3C).

**Fig. 4:**
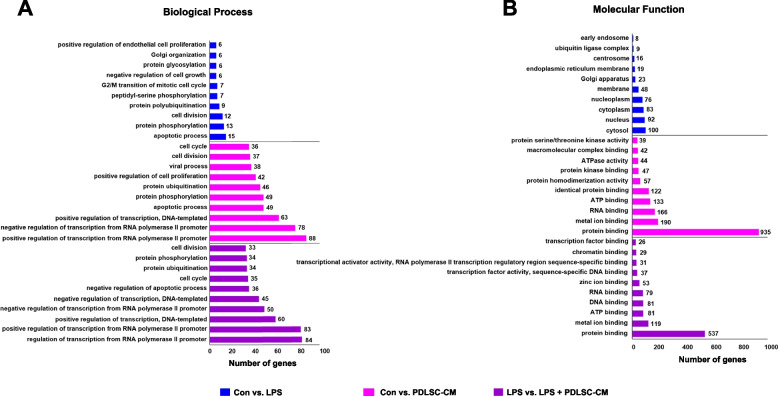
Top 10 enriched GO terms according to (**A**) biological processes and (**B**) molecular functions from the DAVID GO term analysis for the DEGs (*P*‑value < 0.05)

To assess the gene functions, we performed GO enrichment analysis of the DEGs in the different comparison groups. GO terms were arranged according to biological process and molecular function classifications, and the top 10 enriched GO terms were identified. The predominant biological processes included protein phosphorylation and apoptotic process in the control vs. LPS group and transcription regulation in the control vs. PDLSC-CM and LPS vs. LPS + PDLSC-CM groups (Fig. 4A). In the molecular function category, the cell organelle and protein binding terms were enriched in each comparison (Fig. 4B).

### KEGG pathway analysis

**Fig. 5:**
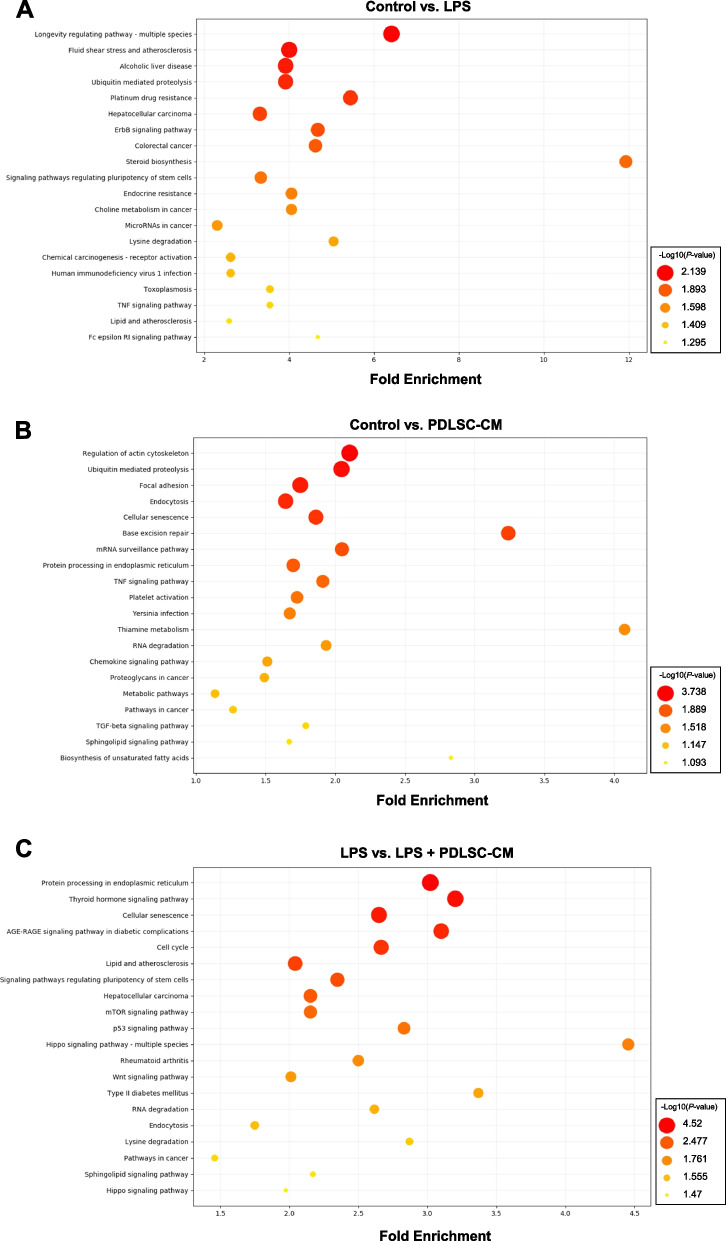
KEGG pathways of DEGs in (**A**) the control vs. LPS, **B** the control vs. PDLSC-CM, and (**C**) the LPS vs. LPS + PDLSC-CM comparison with the top 20 enrichment scores. The size and color of the circle depict − log_10_ (*P*-value). The position of the X-axis represents the fold enrichment

We also analyzed the top 20 KEGG enrichment pathways of the DEGs in the three comparisons, the results of which are presented in Fig. 5. The DEGs in the control vs. LPS group included the ubiquitin-mediated proteolysis, epidermal growth factor receptor family (ErbB) signaling, steroid biosynthesis, tumor necrosis factor (TNF) signaling, and Fc epsilon RI signaling pathways (Fig. 5A). The DEGs in the control vs. PDLSC-CM group were enriched in the endocytosis, base excision repair, regulation of actin cytoskeleton, focal adhesion, and mRNA surveillance pathways (Fig. 5B). The DEGs of the LPS vs. LPS + PDLSC-CM group included the protein processing in the ER, cell cycle, p53 signaling, mammalian target of rapamycin (mTOR) signaling, and Wnt signaling pathways (Fig. 5C).

### Protein–protein interaction (PPI) network construction

**Fig. 6:**
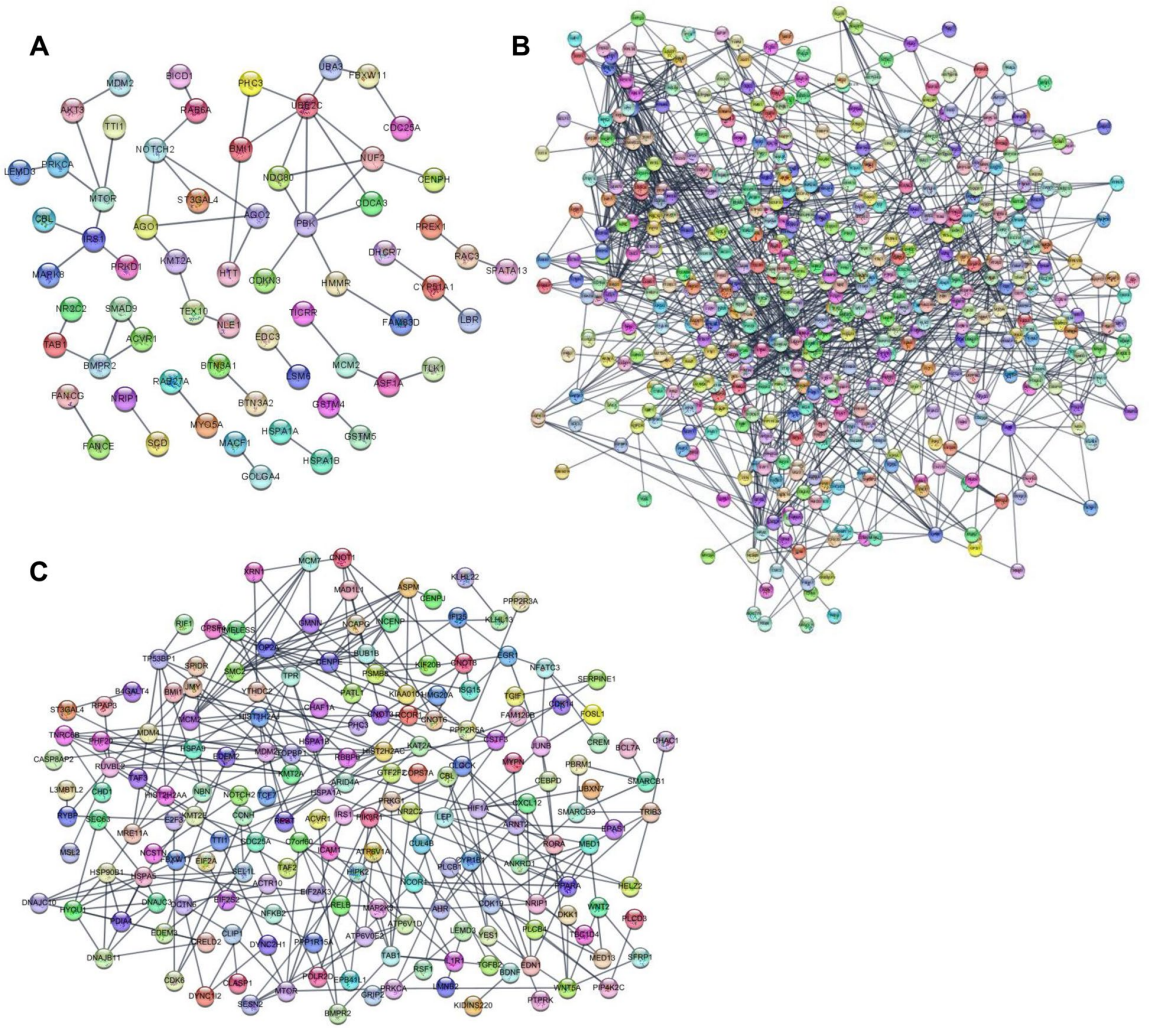
PPI network analysis of DEGs in (**A**) the control vs. LPS, **B** the control vs. PDLSC-CM, **C** the LPS vs. LPS + PDLSC-CM comparison. The PPI network was evaluated using the STRING database. Each node denotes a protein, and each edge depicts an interaction. A confidence (interaction) score of at least 0.9 was considered significant

A functional PPI network was constructed with the DEGs identified using the results of STRING analysis combined with confidence scores. The predicted PPI network in each comparison was mapped with nodes (representing proteins) and edges (representing interactions) (Fig. 6). There were 242 nodes and 58 edges in the control vs. LPS group (Fig. 6A), 1,273 nodes and 1,265 edges in the control vs. PDLSC-CM group (Fig. 6B), and 739 nodes and 354 edges in the LPS vs. LPS + PDLSC-CM group (Fig. 6C). The top 5 hub proteins in each comparison were also selected (Table 2). The proteins with the highest numbers of interactions were ubiquitin-conjugating enzyme E2C (UBE2C) (control vs. LPS group), heat shock protein 90-kDa alpha family class A member 1 (HSP90AA1) (control vs. PDLSC-CM group), and heat shock protein family A (Hsp70) member 5 (HSPA5) (LPS vs. LPS + PDLSC-CM group).

**Table 2:** Top hub proteins with the highest number of interactions

Gene Name	Protein description	#Interacting proteins
**Control vs. LPS**		
UBE2C	ubiquitin conjugating enzyme E2C	7
PBK	PDZ binding kinase	6
NOTCH2	notch 2	4
NUF2	NUF2, NDC80 kinetochore complex component	4
IRS1	insulin receptor substrate 1	4
**Control vs. PDLSC-CM**		
HSP90AA1	heat shock protein 90 kDa α family, member A1	31
RPS5	ribosomal protein S5	27
HRAS	Harvey rat sarcoma viral oncogene homolog	27
BYSL	bystin like	27
NOP58	NOP58 ribonucleoprotein	26
**LPS vs. LPS + PDLSC-CM**		
HSPA5	heat shock protein family A (Hsp70) member 5	11
BUB1B	BUB1 mitotic checkpoint serine /threonine kinase B	9
TPR	translocated promoter region, nuclear basket protein	9
HIST2H2AC	histone cluster 2, H2ac	9
ASPM	abnormal spindle microtubule assembly	8

## Discussion

This study systematically analyzed the protein contents of PDLSC-CM using a proteomics approach and then demonstrated that PDLSC-CM could recapitulate the regenerative potential of PDLSCs under LPS-activated inflammatory conditions through validating the molecular dynamics and the potential signaling pathways by employing transcriptomics. We found that PDLSC-CM included various proteins that are capable of regulating PDLSC bioactivity and stem cell differentiation. Our LC–MS/MS analysis revealed the abundant amount of ECM components including fibronectin and type I collagen, produced in a variety of cells residing in connective tissues, bone, and periodontal tissues [33–35]. These ECM molecules have extensively considered as essential regulators that play a role in various stages of the cell growth, adhesion, and differentiation [36, 37]. In the research challenges in dentistry, fibronectin and type I collagen are expressed and localized in the dental mandibles during embryonic osteogenesis [38]. In addition, these components have shown to function as crucial signaling molecules to promote regeneration and wound healing in periodontal tissue [29, 39]. Our proteomic profile also presented the significant expression of keratin, a cytoskeletal filament, concerned with cell proliferation, adhesion, migration, and cell–cell or cell-ECM contacts [40, 41]. Keratin is a well-known and accepted biomaterial with biocompatibility and physiochemical properties for tissue engineering [42]. Even though dental applications of keratin are still underexplored, recent studies have proved that keratin-modified membranes have shown promising results in pulp-dentin and periodontal tissue regeneration [43, 44]. Thus, based on the key bioactive proteins identified by the proteomic profiling and their regenerative functions, the PDLSC-CM can be provided and further developed for therapeutical alternative in periodontal diseases and periodontium defects. However, further detailed studies are required to determine whether ECM proteins and keratin cytoskeletal filament is the main mechanism encouraging periodontal regeneration by PDLSC-CM treatment.

Characterization of secretome/CM has been well demonstrated as a mixture of a wide range of bioactive proteins and factors to enhance therapeutic and pharmaceutical regenerative therapies, but it is essential to understand that secretome/CM products derived from different stem cell types can present type-specific properties; thus, one secretome/CM may not be effective under all conditions [45]. In this study, we exposed PDLSCs to an inflammatory microenvironment by LPS-preconditioning culture, and we confirmed that the osteogenic differentiation of inflamed PDLSCs was markedly downregulated, but these damaged and weakened PDLSCs recovered their osteogenic differentiation capability when treated with PDLSC-CM. To explore this regenerative capacity of PDLSC-CM, we also determined its secretory functions by transcriptomic approach for the detection and classification of the potential molecular signaling pathways in PDLSCs in response to 1) LPS, 2) PDLSC-CM, or 3) LPS + PDLSC-CM treatment. KEGG pathway analysis of the DEGs identified in the analysis revealed the main pathways involved in each condition. Under the LPS-activated inflammatory condition, we identified enriched signaling pathways, including ubiquitin-mediated proteolysis, TNF signaling, and Fc epsilon RI signaling pathways, which are considerably involved in inflammation and the immune response [46, 47]. Regarding the expression changes induced by PDLSC-CM compared to the control, our results revealed that base excision repair was markedly activated in PDLSCs. Base excision repair is a cellular pathway involved in the repair of damaged DNA and helps to maintain transcriptomic stability [48]. As a DNA repair mechanism is required to restore DNA damage in inflamed tissues, including those present in periodontitis and peri-implantitis lesions [49], introducing PDLSC-CM to diseased oral sites as a treatment may be able to prevent inflammation and tissue degradation. Another important signaling pathway influenced by PDLSC-CM involves the actin cytoskeleton, which generally influences diverse cellular functions, including communication with the ECM [50, 51]. Our previous study suggested that actin cytoskeleton dynamics are among the key signaling pathways involved in enhancing the osteogenic potential of PDLSCs [24]. Several studies have also reported the significant impact of the mechanisms of the actin cytoskeleton in the osteogenic commitment of MSCs and osteogenic cells [52, 53]. As regeneration of periodontitis-induced bone defects is a key challenge in clinical dentistry, PDLSC-CM and its regenerative factors may be potential candidates for encouraging the osteogenic differentiation capacity of osteoprogenitors/stem cells and promoting periodontal tissue regeneration. Lastly, the KEGG pathways identified in the LPS vs. LPS + PDLSC-CM comparison included the mTOR and Wnt signaling pathways, which are involved in various cellular processes and functions, particularly stem cell proliferation and differentiation. Increasing evidence indicates that the activated PI3K/AKT/mTOR pathway restores the osteogenic differentiation of PDLSCs under inflammatory conditions [54, 55]. Profiling the gene expression levels of PDLSCs under certain pharmacological conditions also demonstrated that the self-renewal and osteogenic differentiation capabilities of PDLSCs were upregulated through activating the mTOR signaling pathway [56]. Additionally, it has been well established that PDL tissue responds to Wnt signaling in the homeostasis of the PDL and periodontal tissue formation [57, 58]. We and others previously demonstrated that activation of the Wnt pathway is required for the commitment of PDLSCs to an osteogenic lineage [22, 59, 60]. The experimental evidence in this study links the finding that PDLSC-CM can rehabilitate the inflammation-mediated impairment of PDLSC functions and suggests a novel therapeutic strategy to enhance endogenous periodontal cell/tissue healing and regeneration.

Transcriptomic studies eventually predicted PPI networks and identified differentially abundant proteins in each comparison group. In LPS-activated inflamed PDLSCs, a significant hub protein identified was ubiquitin-conjugating enzyme, which is associated with the regulation of inflammation and the innate immune response; thus, this enzyme has been emphasized as a potential therapeutic target in human health and diseases [61]. In the control vs. PDLSC-CM comparison, HSP90AA1 was identified as a highly interacting and abundant protein. HSP90AA1 has been implicated in the activation of regulator proteins involved in signal transduction, cell cycle regulation, and differentiation [62]. Previous transcriptomic analysis also revealed that HSP90AA1 upregulation is involved in the anti-apoptotic or proliferative effect on human PDLSCs [63]. The PPI network of the LPS vs. LPS + PDLSC-CM group revealed that the protein with the highest number of interactions was HSPA5, also known as GRP78 (glucose-regulatory protein 78). HSPA5 is an essential regulator of ER homeostasis and plays multiple functional roles in cell viability, proliferation, apoptosis, and the immune system [64]. A recent study established that GRP78 (HSPA5) overexpression in PDLSCs stimulates their osteogenic differentiation and mineralization, suggesting that this protein may be a promising therapeutic target for the regeneration and repair of damage to the periodontium induced by periodontal inflammation [65]. According to the identified signaling pathways and protein networks by the transcriptomic analysis, the PDLSC-CM may provide therapeutical advantages for regenerative medicine. However, further studies to examine the significance of each component and a single pathway in regulating PDLSC-CM-induced periodontal regeneration are required.

## Conclusion

Combined with findings from other recent studies, our proteomic and transcriptomic profiling results serve as another translational study providing notable insights into the secretory functions of PDLSC-CM in the recovery of PDLSCs under an inflammatory environment. The results of this study may ultimately serve as a resource to advance cell-free regenerative and therapeutic approaches involving PDLSC-derived secretome/CM in the treatment of periodontal disease.

### Supplementary Information

**Supplementary Material 1.**

**Supplementary Material 2.**

**Supplementary Material 3.**

**Supplementary Material 4.**

**Supplementary Material 5.**

**Supplementary Material 6.**

## Data Availability

The sequence data supporting the findings of this study are available within the paper and its supplementary information files. Scanned images of X-film for western blots are also provided within the supplementary information files.

## References

[1] Suman S, Domingues A, Ratajczak J, Ratajczak MZ. Potential Clinical Applications of Stem Cells in Regenerative Medicine. Adv Exp Med Biol. 2019;1201:1-22. 10.1007/978-3-030-31206-0_1 31898779

[2] Rombouts W, Ploemacher RE (2003). Primary murine MSC show highly efficient homing to the bone marrow but lose homing ability following culture. Leukemia.

[3] Pires AO, Mendes-Pinheiro B, Teixeira FG, Anjo SI, Ribeiro-Samy S, Gomes ED, Serra SC, Silva NA, Manadas B, Sousa N (2016). Unveiling the differences of secretome of human bone marrow mesenchymal stem cells, adipose tissue-derived stem cells, and human umbilical cord perivascular cells: a proteomic analysis. Stem Cells Dev.

[4] Asgarpour K, Shojaei Z, Amiri F, Ai J, Mahjoubin-Tehran M, Ghasemi F, ArefNezhad R, Hamblin MR, Mirzaei H (2020). Exosomal microRNAs derived from mesenchymal stem cells: cell-to-cell messages. Cell Commun Signal.

[5] Harrell CR, Fellabaum C, Jovicic N, Djonov V, Arsenijevic N, Volarevic V (2019). Molecular mechanisms responsible for therapeutic potential of mesenchymal stem cell-derived secretome. Cells.

[6] Nagata M, Iwasaki K, Akazawa K, Komaki M, Yokoyama N, Izumi Y, Morita I (2017). Conditioned medium from periodontal ligament stem cells enhances periodontal regeneration. Tissue Eng Part A.

[7] Rajan TS, Scionti D, Diomede F, Grassi G, Pollastro F, Piattelli A, Cocco L, Bramanti P, Mazzon E, Trubiani O (2017). Gingival stromal cells as an in vitro model: Cannabidiol modulates genes linked with amyotrophic lateral sclerosis. J Cell Biochem.

[8] Sedgley CM, Botero TM (2012). Dental stem cells and their sources. Dent Clin.

[9] Achilleos A, Trainor PA (2012). Neural crest stem cells: discovery, properties and potential for therapy. Cell Res.

[10] Rajan TS, Giacoppo S, Trubiani O, Diomede F, Piattelli A, Bramanti P, Mazzon E (2016). Conditioned medium of periodontal ligament mesenchymal stem cells exert anti-inflammatory effects in lipopolysaccharide-activated mouse motoneurons. Exp Cell Res.

[11] Qiu J, Wang X, Zhou H, Zhang C, Wang Y, Huang J, Liu M, Yang P, Song A (2020). Enhancement of periodontal tissue regeneration by conditioned media from gingiva-derived or periodontal ligament-derived mesenchymal stem cells: a comparative study in rats. Stem Cell Res Ther.

[12] Gugliandolo A, Diomede F, Pizzicannella J, Chiricosta L, Trubiani O, Mazzon E (2022). Potential anti-inflammatory effects of a new lyophilized formulation of the conditioned medium derived from periodontal ligament stem cells. Biomedicines.

[13] Beatty LA, Mansour KL, Bryant EJ, Garcia-Godoy F, Santos Pantaleon D, Sawatari Y, Huang C, Garcia-Godoy F (2022). Chondroprotective effects of periodontal ligament-derived stem cells conditioned medium on articular cartilage after impact injury. Stem Cells Dev.

[14] Mizuno N, Ozeki Y, Shiba H, Kajiya M, Nagahara T, Takeda K, Kawaguchi H, Abiko Y, Kurihara H (2008). Humoral factors released from human periodontal ligament cells influence calcification and proliferation in human bone marrow mesenchymal stem cells. J Periodontol.

[15] Novello S, Tricot-Doleux S, Novella A, Pellen-Mussi P, Jeanne S (2022). Influence of periodontal ligament stem cell-derived conditioned medium on osteoblasts. Pharmaceutics.

[16] Giacoppo S, Thangavelu SR, Diomede F, Bramanti P, Conti P, Trubiani O, Mazzon E (2017). Anti-inflammatory effects of hypoxia-preconditioned human periodontal ligament cell secretome in an experimental model of multiple sclerosis: a key role of IL-37. FASEB J.

[17] Iwasaki K, Akazawa K, Nagata M, Komaki M, Peng Y, Umeda M, Watabe T, Morita I (2021). Angiogenic effects of secreted factors from periodontal ligament stem cells. Dent J.

[18] Bousnaki M, Bakopoulou A, Pich A, Papachristou E, Kritis A, Koidis P (2022). Mapping the secretome of dental pulp stem cells under variable microenvironmental conditions. Stem Cell Rev Rep.

[19] Huang C, Vesvoranan O, Yin X, Montoya A, Londono V, Sawatari Y, Garcia-Godoy F (2021). Anti-inflammatory effects of conditioned medium of periodontal ligament-derived stem cells on chondrocytes, synoviocytes, and meniscus cells. Stem Cells Dev.

[20] Ge S, He W, Zhang L, Lin S, Luo Y, Chen Q, Zeng M (2022). Ghrelin pretreatment enhanced the protective effect of bone marrow-derived mesenchymal stem cell-conditioned medium on lipopolysaccharide-induced endothelial cell injury. Mol Cell Endocrinol.

[21] Zhang Z, Sheng H, Liao L, Xu C, Zhang A, Yang Y, Zhao L, Duan L, Chen H, Zhang B (2020). Mesenchymal stem cell-conditioned medium improves mitochondrial dysfunction and suppresses apoptosis in okadaic acid-treated SH-SY5Y cells by extracellular vesicle mitochondrial transfer. J Alzheimers Dis.

[22] Shim NY, Ryu J, Heo JS (2022). Osteoinductive function of fucoidan on periodontal ligament stem cells. Role of PI3K/Akt and Wnt/β-catenin signaling pathways. Oral Dis.

[23] Nam O, Park J, Lee H, Jin E (2019). De novo transcriptome profile of coccolithophorid alga Emiliania huxleyi CCMP371 at different calcium concentrations with proteome analysis. PLoS ONE.

[24] Kwack KH, Ji JY, Park B, Heo JS (2022). Fucoidan (Undaria pinnatifida)/polydopamine composite-modified surface promotes osteogenic potential of periodontal ligament stem cells. Mar Drugs.

[25] Langmead B, Salzberg SL (2012). Fast gapped-read alignment with Bowtie 2. Nat Methods.

[26] Quinlan AR, Hall IM (2010). BEDTools: a flexible suite of utilities for comparing genomic features. Bioinformatics.

[27] Gentleman RC, Carey VJ, Bates DM, Bolstad B, Dettling M, Dudoit S, Ellis B, Gautier L, Ge Y, Gentry J (2004). Bioconductor: open software development for computational biology and bioinformatics. Genome Biol.

[28] Felgueiras HP, Evans MD, Migonney V (2015). Contribution of fibronectin and vitronectin to the adhesion and morphology of MC3T3-E1 osteoblastic cells to poly (NaSS) grafted Ti6Al4V. Acta Biomater.

[29] Imber JC, Roccuzzo A, Stähli A, Saulacic N, Deschner J, Sculean A, Bosshardt DD (2021). Immunohistochemical evaluation of periodontal regeneration using a porous collagen scaffold. Int J Mol Sci.

[30] Janjić K, Agis H, Moritz A, Rausch-Fan X, Andrukhov O (2022). Effects of collagen membranes and bone substitute differ in periodontal ligament cell microtissues and monolayers. J Periodontol.

[31] Wu YL, Lin CW, Cheng NC, Yang KC, Yu J (2017). Modulation of keratin in adhesion, proliferation, adipogenic, and osteogenic differentiation of porcine adipose-derived stem cells. J Biomed Mater Res B Appl Biomater.

[32] Chen WY, Li X, Feng Y, Lin S, Peng L, Huang D (2020). M-keratin nano-materials create a mineralized micro-circumstance to promote proliferation and differentiation of DPSCs. J Mater Sci Mater Med.

[33] Medina-Ortiz WE, Belmares R, Neubauer S, Wordinger RJ, Clark AF (2013). Cellular fibronectin expression in human trabecular meshwork and induction by transforming growth factor-β2. Invest Ophthalmol Vis Sci.

[34] Parkar MH, Bakalios P, Newman HN, Olsen I (1997). Expression and splicing of the fibronectin gene in healthy and diseased periodontal tissue. Eur J Oral Sci.

[35] Ho SP, Kurylo MP, Fong TK, Lee SS, Wagner HD, Ryder MI, Marshall GW (2010). The biomechanical characteristics of the bone-periodontal ligament-cementum complex. Biomaterials.

[36] Wang Z, Wang Z, Pang Y, Tong H, Yan Y, Li S, Li S (2020). Fibronectin type III domain-containing 4 promotes the migration and differentiation of bovine skeletal muscle-derived satellite cells via focal adhesion kinase. Cell Adh Migr.

[37] Sugiaman VK, Djuanda R, Pranata N, Naliani S, Demolsky WL, Jeffrey (2022). Tissue engineering with Stem Cell from Human Exfoliated Deciduous Teeth (SHED) and collagen matrix, regulated by growth factor in regenerating the dental pulp. Polymers (Basel).

[38] Sasano Y, Li HC, Zhu JX, Imanaka-Yoshida K, Mizoguchi I, Kagayama M (2000). Immunohistochemical localization of type I collagen, fibronectin and tenascin C during embryonic osteogenesis in the dentary of mandibles and tibias in rats. Histochem J.

[39] Shirbhate U, Bajaj P, Pandher J, Durge K (2022). Fibronectin and its applications in dentistry and periodontics: a cell behaviour conditioner. Cureus.

[40] Zhang X, Yin M, Zhang LJ (2019). Keratin 6, 16 and 17-critical barrier alarmin molecules in skin wounds and psoriasis. Cells.

[41] Yoon S, Leube RE (2019). Keratin intermediate filaments: intermediaries of epithelial cell migration. Essays Biochem.

[42] Feroz S, Muhammad N, Ranayake J, Dias G (2020). Keratin - Based materials for biomedical applications. Bioact Mater.

[43] Sharma LA, Ramesh N, Sharma A, Ratnayake JTB, Love RM, Alavi SE, Wilson MJ, Dias GJ (2023). In vitro effects of wool-derived keratin on human dental pulp-derived stem cells for endodontic applications. Br J Oral Maxillofac Surg.

[44] Atrian M, Kharaziha M, Javidan H, Alihosseini F, Emadi R (2022). Zwitterionic keratin coating on silk-Laponite fibrous membranes for guided bone regeneration. J Tissue Eng Regen Med.

[45] Flower T, Pulsipher V, Moreno A (2015). A new tool in regenerative medicine: mesenchymal stem cell secretome. J Stem Cell Res Ther.

[46] Wang LM, Zhao N, Zhang J, Sun QF, Yang CZ, Yang PS (2018). Tumor necrosis factor-alpha inhibits osteogenic differentiation of pre-osteoblasts by downregulation of EphB4 signaling via activated nuclear factor-kappaB signaling pathway. J Periodont Res.

[47] Kim Y, Hur G, Lee SW, Lee S, Lee S, Kim S, Rho M (2020). AGK2 ameliorates mast cell-mediated allergic airway inflammation and fibrosis by inhibiting FcεRI/TGF-β signaling pathway. Pharmacol Res.

[48] Demin AA, Hirota K, Tsuda M, Adamowicz M, Hailstone R, Brazina J, Gittens W, Kalasova I, Shao Z, Zha S (2021). XRCC1 prevents toxic PARP1 trapping during DNA base excision repair. Mol Cell..

[49] Dionigi C, Larsson L, Carcuac O, Berglundh T (2020). Cellular expression of DNA damage/repair and reactive oxygen/nitrogen species in human periodontitis and peri-implantitis lesions. J Clin Periodontol.

[50] Case LB, Waterman CM (2015). Integration of actin dynamics and cell adhesion by a three-dimensional, mechanosensitive molecular clutch. Nat Cell Biol.

[51] Schwarz US, Gardel ML (2012). United we stand–integrating the actin cytoskeleton and cell–matrix adhesions in cellular mechanotransduction. J Cell Sci.

[52] Zhao Y, Sun Q, Wang S, Huo B (2019). Spreading shape and area regulate the osteogenesis of mesenchymal stem cells. Tissue Eng Regen Med.

[53] Vishavkarma R, Raghavan S, Kuyyamudi C, Majumder A, Dhawan J, Pullarkat PA (2014). Role of actin filaments in correlating nuclear shape and cell spreading. PLoS ONE.

[54] Zhao X, Sun W, Guo B, Cui L (2022). Circular RNA BIRC6 depletion promotes osteogenic differentiation of periodontal ligament stem cells via the miR-543/PTEN/PI3K/AKT/mTOR signaling pathway in the inflammatory microenvironment. Stem Cell Res Ther.

[55] Xu X, He X, Wang J, Li X, Xia Y, Tan Y, Chen F (2019). Role of the P2X7 receptor in inflammation-mediated changes in the osteogenesis of periodontal ligament stem cells. Cell Death Dis.

[56] Arora P, Li W, Huang X, Yu W, Huang R, Jiang Q, Chen C (2022). Metabolic reconfiguration activates stemness and immunomodulation of PDLSCs. Int J Mol Sci.

[57] Lim WH, Liu B, Cheng D, Williams BO, Mah SJ, Helms JA (2014). Wnt signaling regulates homeostasis of the periodontal ligament. J Periodont Res.

[58] Zhong Z, Zylstra-Diegel CR, Schumacher CA, Baker JJ, Carpenter AC, Rao S, Yao W, Guan M, Helms JA, Lane NE (2012). Wntless functions in mature osteoblasts to regulate bone mass. Proc Natl Acad Sci.

[59] Ying M, Zhang B (2023). Daidzein promotes the proliferation and osteogenic differentiation of periodontal ligament stem cell. Oral Dis.

[60] Cui Q, Li N, Nie F, Yang F, Li H, Zhang J (2021). Vitamin K2 promotes the osteogenic differentiation of periodontal ligament stem cells via the Wnt/β-catenin signaling pathway. Arch Oral Biol.

[61] Liu R, Cheng Q, Song X, Wang H, Wang X, Wang L, Zhu B, Song L (2019). A vital ubiquitin-conjugating enzyme CgUbe2g1 participated in regulation of immune response of Pacific oyster Crassostrea gigas. Dev Comp Immunol.

[62] Ma D, Cui L, Gao J, Yan W, Liu Y, Xu S, Wu B (2014). Proteomic analysis of mesenchymal stem cells from normal and deep carious dental pulp. PLoS ONE.

[63] Lanza Cariccio V, Scionti D, Raffa A, Iori R, Pollastro F, Diomede F, Bramanti P, Trubiani O, Mazzon E (2018). Treatment of periodontal ligament stem cells with MOR and CBD promotes cell survival and neuronal differentiation via the PI3K/Akt/mTOR pathway. Int J Mol Sci.

[64] Conner C, Lager TW, Guldner IH, Wu M, Hishida Y, Hishida T, Ruiz S, Yamasaki AE, Gilson RC, Belmonte JCI (2020). Cell surface GRP78 promotes stemness in normal and neoplastic cells. Sci Rep.

[65] Merkel A, Chen Y, Villani C, George A (2023). GRP78 promotes the osteogenic and angiogenic response in periodontal ligament stem cells. Eur Cell Mater.

