# Supplementary material for: Translating proteome and transcriptome dynamics of periodontal ligament stem cell-derived secretome/conditioned medium in an in vitro model of periodontitis
Source: BMC Oral Health. 2024 Mar 27;24:390. doi: 10.1186/s12903-024-04167-z (PMC10967149; doi:10.1186/s12903-024-04167-z)
Supplement: Supplementary file 5 — Supplementary Material 5. [file 12903_2024_4167_MOESM5_ESM.pptx]

## Slide 1
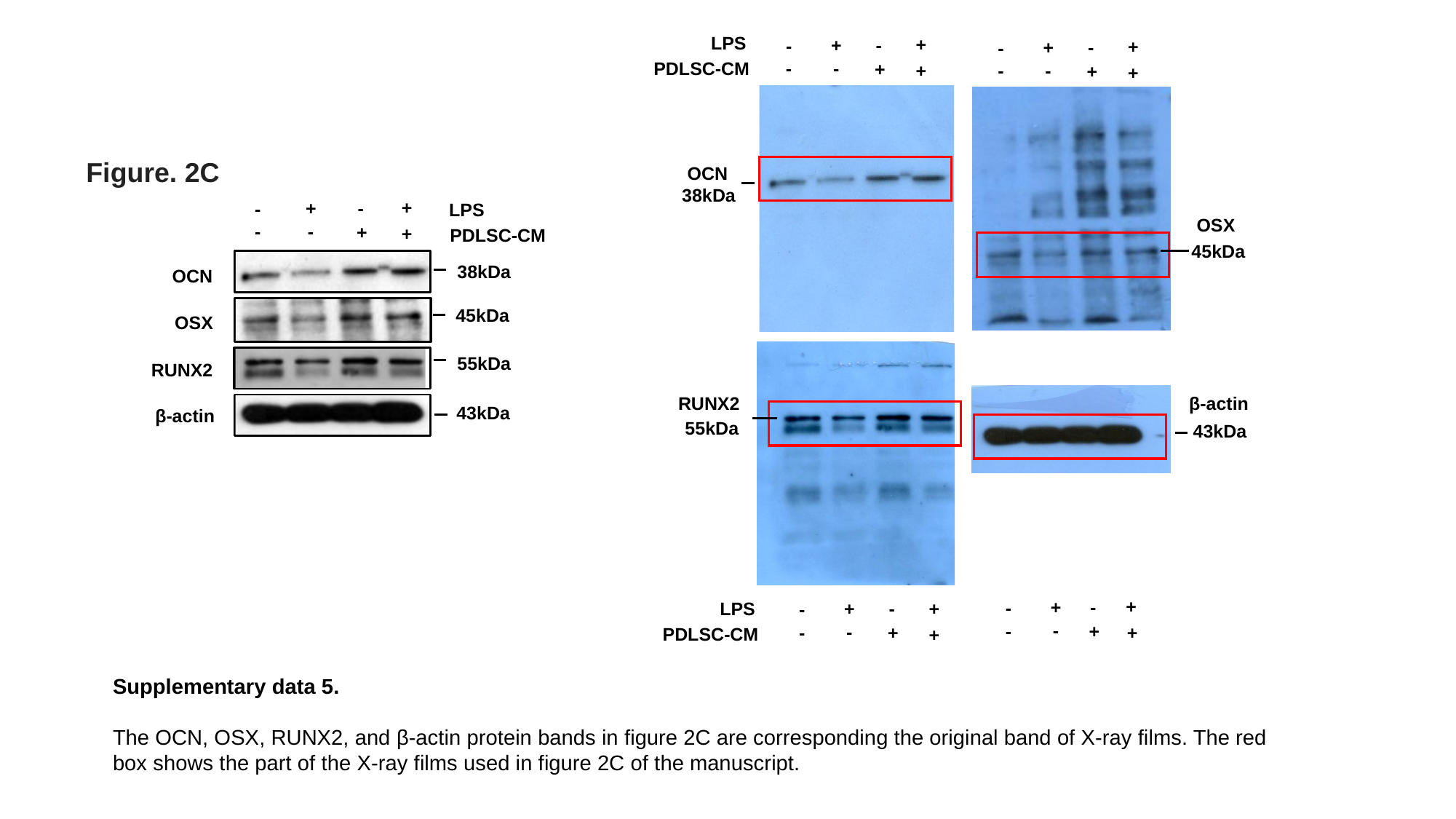

LPS
PDLSC-CM
+
+
-
-
-
-
+
+
+
+
-
-
-
-
+
+
Figure. 2C
OCN
38kDa
+
+
-
-
LPS
-
-
+
+
PDLSC-CM
38kDa
OCN
45kDa
OSX
55kDa
RUNX2
43kDa
β-actin
OSX
45kDa
RUNX2
β-actin
55kDa
43kDa
+
+
-
-
-
-
+
+
+
+
-
-
-
-
+
+
LPS
PDLSC-CM
Supplementary data 5.
The OCN, OSX, RUNX2, and β-actin protein bands in figure 2C are corresponding the original band of X-ray films. The red box shows the part of the X-ray films used in figure 2C of the manuscript.
