# Supplementary figures and images for: Translating proteome and transcriptome dynamics of periodontal ligament stem cell-derived secretome/conditioned medium in an in vitro model of periodontitis
Source: BMC Oral Health. 2024 Mar 27;24:390. doi: 10.1186/s12903-024-04167-z (PMC10967149; doi:10.1186/s12903-024-04167-z)

## Schematic workflow

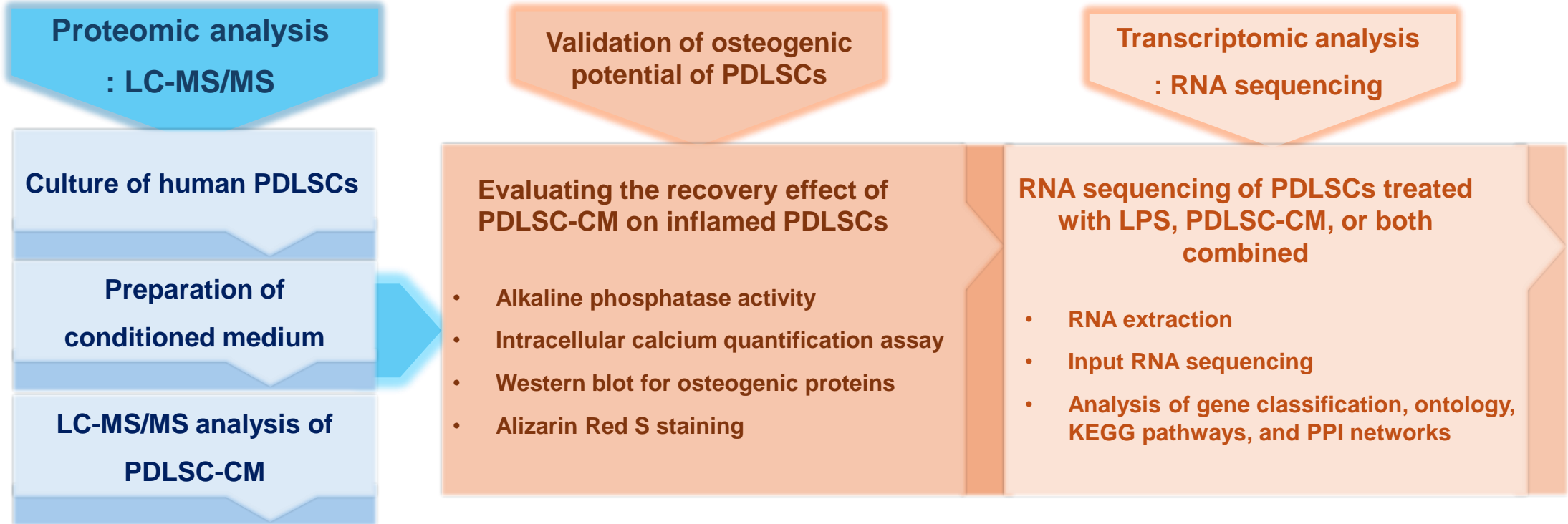

Supplement: Supplementary file 6 — Supplementary Material 6. [file 12903_2024_4167_MOESM6_ESM.pdf]
